# Use of Immunization Information Systems in Ascertainment of COVID-19 Vaccinations for Claims-Based Vaccine Safety and Effectiveness Studies

**DOI:** 10.1001/jamanetworkopen.2023.13512

**Published:** 2023-05-16

**Authors:** Karen L. Schneider, Elizabeth J. Bell, C. K. Zhou, Grace Yang, Patricia Lloyd, Tainya C. Clarke, Michael Wilkinson, Emily E. Myers, Kandace L. Amend, John D. Seeger, Yoganand Chillarige, Richard A. Forshee, Azadeh Shoaibi, Steven A. Anderson, Hui-Lee Wong

**Affiliations:** 1 OptumServe Consulting, Falls Church, Virginia; 2 Optum Epidemiology, Boston, Massachusetts; 3 Clinical Safety and Risk Management, Moderna, Cambridge, Massachusetts; 4 Center for Biologics Evaluation and Research, US Food and Drug Administration, Silver Spring, Maryland; 5 Acumen, Burlingame, California

## Abstract

**Importance:**

Safety and effectiveness studies of COVID-19 vaccines are being conducted using clinical data, including administrative claims. However, claims data only partially capture administered COVID-19 vaccine doses for numerous reasons, such as vaccination at sites that do not generate claims for reimbursement.

**Objective:**

To evaluate the extent to which Immunization Information Systems (IIS) data linked to claims data enhances claims-based COVID-19 vaccine capture for a commercially insured population and to estimate the magnitude of misclassification of vaccinated individuals as having unvaccinated status in the linked IIS and claims data.

**Design, Setting, and Participants:**

This cohort study used claims data from a commercial health insurance database and obtained vaccination data from IIS repositories in 11 US states. Participants were individuals younger than 65 years who resided in 1 of 11 states of interest and who were insured in health plans from December 1, 2020, through December 31, 2021.

**Main Outcomes and Measures:**

Estimated proportion of individuals with at least 1 dose of any COVID-19 vaccine and proportion of individuals with a completed vaccine series based on general population guidelines. Vaccination status estimates were calculated and compared using claims data alone and linked IIS and claims data. Remaining misclassification of vaccination status was assessed by comparing linked IIS and claims data estimates with estimates from external surveillance data sources (Centers for Disease Control and Prevention [CDC] and state Department of Health [DOH]) and capture-recapture analysis.

**Results:**

This cohort study included 5 112 722 individuals (mean [SD] age, 33.5 [17.6] years; 2 618 098 females [51.2%]) from 11 states. Characteristics of those who received at least 1 vaccine dose and those who completed a vaccine series were similar to the overall study population. The proportion with at least 1 vaccine dose increased from 32.8% using claims data alone to 48.1% when the data were supplemented with IIS vaccination records. Vaccination estimates using linked IIS and claims data varied widely by state. The percentage of individuals who completed a vaccine series increased from 24.4% to 41.9% after the addition of IIS vaccine records and varied across states. The percentages of underrecording using linked IIS and claims data were 12.1% to 47.1% lower than those using CDC data, 9.1% to 46.9% lower than the state DOH, and 9.2% to 50.9% lower than capture-recapture analysis.

**Conclusion and Relevance:**

Results of this study suggested that supplementing COVID-19 claims records with IIS vaccination records substantially increased the number of individuals who were identified as vaccinated, yet potential underrecording remained. Improvements in reporting vaccination data to IIS infrastructures could allow frequent updates of vaccination status for all individuals and all vaccines.

## Key Points

**Question:**

Does the addition of Immunization Information System (IIS) data enhance COVID-19 vaccine ascertainment in commercial health care claims data in the US?

**Findings:**

In this cohort study of 5 112 722 individuals, the percentage of persons with at least 1 COVID-19 vaccine dose increased from 32.8% using claims data alone to 48.1% after linking claims data to IIS vaccination records; a similar increase was seen for estimates of individuals who completed a vaccine series.

**Meaning:**

Findings of this study suggest that linkage of claims data to IIS vaccination records was associated with increased COVID-19 vaccine ascertainment and that improvements in reporting vaccination data to IIS infrastructures allow frequent updates of vaccination status for all individuals and all vaccines.

## Teaser

This cohort study assesses whether adding Immunization Information System vaccination data to administrative claims data can help improve ascertainment of COVID-19 vaccination status among commercially insured US adults.

## Introduction

The US Food and Drug Administration’s (FDA’s) Biologics Effectiveness and Safety (BEST) Initiative conducts active surveillance of biologic products, such as vaccines. This initiative includes the design and conduct of safety and effectiveness studies of the COVID-19 vaccines using clinical data from a variety of sources, including administrative claims.

Accurate COVID-19 vaccination data are necessary for safety and effectiveness studies of COVID-19 vaccines.^1,2^ Health insurance claims alone incompletely capture vaccine administration and thereby vaccination status. For example, vaccines administered in some settings (eg, mass vaccination campaigns) do not necessarily generate claims. The Centers for Medicare and Medicaid Services found that Medicare claims identified only 17.5 million individuals 65 years or older with at least 1 dose of COVID-19 vaccine compared with the 44.1 million vaccinated individuals estimated by the Centers for Disease Control and Prevention (CDC).^3,4^ For studies comparing vaccinated to unvaccinated people, misclassification of vaccination status could lead to biased effect size estimates.

The FDA’s Center for Biologics Evaluation and Research (CBER) examined whether administrative claims data of a commercially insured population could be linked to the Immunization Information Systems (IIS) to enhance claims-based capture of COVID-19 vaccination status. Immunization Information Systems databases are centralized, population-based computerized repositories of vaccine records that theoretically include all doses administered by participating vaccination providers in a given area, regardless of whether these providers submitted billing claims.^4^ Specifically, this CBER study aimed (1) to evaluate the extent to which IIS data linked to claims data enhances claims-based COVID-19 vaccine capture for a commercially insured population and (2) to estimate the magnitude of misclassification of vaccinated individuals as having unvaccinated status in linked IIS and claims data. Results from the initial study are available on the BEST Initiative website.^5^

## Methods

As a Congressionally mandated public health surveillance activity within the BEST Initiative, this observational, retrospective cohort study was not subject to the Common Rule as verified by the Office for Human Research Protections and thus exempt from institutional review board review and approval. All IIS repositories involved in this study allowed the use of their COVID-19 vaccine data for public health surveillance activities. We followed the Strengthening the Reporting of Observational Studies in Epidemiology (STROBE) reporting guideline.

### Data Sources

A linked IIS and claims database was the main data source for this cohort study. This database was developed by supplementing a claims database, the Optum Research Database (Optum Inc), with COVID-19 vaccination records from IIS repositories.

The Optum Research Database is a geographically diverse US database that contains medical, immunization, laboratory test orders and results, and prescription drug claims for more than 250 million unique members with commercial insurance or Medicare Advantage coverage. A variation of this database combines preadjudicated commercial medical (hospital and physician) claims with linked enrollment and adjudicated commercial pharmacy claims. Preadjudicated medical claims (chosen over adjudicated for their shorter lag time) undergo initial daily processing from clinicians and health care facilities across the US who accept the insurance plans included in the claims data. The preadjudicated medical claims have approximately 90% completeness at 2 months for inpatient claims and more than 70% completeness at 1 month for outpatient claims. Pharmacy claims are adjudicated at the point of sale in real time. Pharmacy claims used for this study were refreshed on January 18, 2022, and preadjudicated medical claims were refreshed on January 15, 2022.

Different IIS repositories were requested to link their COVID-19 vaccination data to individuals in the claims database. At the time of this study, 14 IIS repositories in 11 states representing all 4 census regions responded (state names were masked because the purpose of this study was to assess the added benefit of enhancing claims data with IIS data and not to compare vaccination capture across states). Jurisdictions were added as available, increasing both the sample size and generalizability to the US commercially insured population. The IIS data were refreshed in the IIS repositories at various intervals (eTable 1 in Supplement 1). Each IIS repository used an IIS-specific linkage algorithm to match individuals to COVID-19 vaccines that were administered in their jurisdiction, and the median IIS match percentage was calculated by dividing the individual count in the claims database by the number of records returned from the IIS database (eTable 2 in Supplement 1). The IIS data included vaccination data reported by a variety of vaccination providers, including physicians, retail pharmacies, hospitals, local public health departments, and mass vaccination campaigns within the jurisdiction.^4^ Vaccination information was shared via a real-time electronic interface between the vaccination provider’s electronic system and the IIS repository or was entered directly into the IIS user interface.

Vaccination records were deduplicated within each data source (ie, claims and IIS) and then across data sources by insured individual’s ID, date of service, and vaccine type. When deduplicating records, priority was given to records derived from claims. The remaining vaccination records were used to create the linked IIS and claims vaccine indicator variable (eTable 3 in Supplement 1).

### Study Population and Vaccination Status

The study population included individuals younger than 65 years who resided in 1 of the 11 states of interest. These individuals were commercially insured by health plans in the claims database at any time during the study period (ie, between December 1, 2020 [month that the first COVID-19 vaccine received emergency use authorization from the FDA], and December 31, 2021).

Receipt of the BNT162b2 (Pfizer/BioNTech), mRNA-1273 (Moderna), or Ad26.COV2.S (Janssen) COVID-19 vaccine was identified with CVX (vaccine administered) codes used by IIS databases, *Current Procedural Terminology* codes, Healthcare Common Procedure Coding System codes, National Drug Codes, and *International Statistical Classification of Diseases and Related Health Problems, Tenth Revision* procedure codes during the study period (codes are provided in eTable 3 in Supplement 1). Number of doses was determined using the date of vaccine administration. The first dose was defined as the vaccine record with the earliest administration date, and the second dose was defined as the second record with an administration date at least 3 days after the first dose. We excluded doses that were administered less than 3 days after the first dose.

For this study, vaccination status was defined as having received at least 1 dose of a COVID-19 vaccine regardless of vaccine brand or type or completed a vaccine series, depending on vaccine brand or type. If the vaccine received was Ad26.COV2.S (Janssen), then only 1 vaccine record was required to have a completed vaccine series. For the purposes of this study, if the vaccine received was BNT162b2 (Pfizer/BioNTech), mRNA-1273 (Moderna), a combination of BNT162b2 and mRNA-1273, or unknown vaccine brand or type, we defined a completed vaccine series as presence of at least 2 vaccine records that were at least 3 days apart.

### Statistical Analysis

We conducted descriptive analyses of the characteristics (ie, state of residence, age, and sex [eTable 4 in Supplement 1] and vaccine type) of the overall study population, those who received at least 1 vaccine dose, and those who completed a vaccine series. Sex (male or female) was self-reported by individuals at the time of health plan enrollment. Vaccination counts by date of administration were also calculated. Race and ethnicity data were not available in claims data.

To address the first study objective, we summarized the number and proportion of the study population (overall and by state of residence) with at least 1 vaccine dose or with a completed vaccine series using claims data only and linked IIS and claims data. To address the second study objective, we estimated the magnitude of misclassification in vaccination estimates by comparing the linked IIS and claims data estimates for the commercial population to (1) age-standardized external references from the CDC and state Department of Health (DOH) for the general population (with commercial insurance, Medicaid and Medicare coverage, or no insurance) and (2) data-driven estimates based on capture-recapture analyses^6^ (eMethods 1 in Supplement 1 provides details on age standardization; eMethods 2 in Supplement 1 provides details on the capture-recapture method). The capture-recapture analysis used counts from 2 independent data sources (IIS and claims databases) to estimate the number of individuals who were not captured by either of the data sources. Specifically, these CDC, state DOH, and capture-recapture–adjusted vaccination estimates were compared with the linked IIS and claims data estimates for the commercially insured population by calculating the remaining underrecording of vaccination in the linked IIS and claims database (ie, the proportion of vaccinated individuals who were misclassified as unvaccinated) at the state level. We calculated underrecording of vaccination as the difference between the vaccination estimate from each of these sources and the linked IIS and claims data estimate divided by the vaccination estimate from the other source. It can be interpreted as a percentage change from the CDC, state DOH, or capture-recapture–adjusted estimates.

We performed 2 sensitivity analyses. First, for the main analysis, we required only 1 day of medical or pharmacy coverage during the study period, which had the potential to overestimate the mismatch between claims and IIS data because individuals may have received a vaccine after disenrollment from health insurance. To address this scenario, we restricted the analysis to individuals with continuous enrollment during the study period (unless disenrollment was due to death) to align the opportunity for vaccine ascertainment in claims and IIS databases. Second, although claims were available for services sought inside and outside the individual’s state of residence, vaccination would not be recorded if received outside of the state that did not result in a claim (eg, at a mass vaccination site), with the exception of states with agreements to share IIS data with other states and individuals reporting the vaccination to a vaccination provider in their home state and the provider submitting the information to the IIS repository. To assess the impact of this scenario, we calculated vaccination estimates that were restricted to individuals with all claims arising from a single state. All analyses were performed in SAS Enterprise Guide, version 6.1 (SAS Institute Inc).

## Results

Characteristics of the overall study population, individuals who received at least 1 vaccine dose, and individuals who completed a vaccine series are detailed in eTables 5 and 6 in Supplement 1. Briefly, characteristics were similar across the overall study population and the 2 subsets except for age, which may be explained by variation in IIS database refresh dates (eTable 1 in Supplement 1) and when the vaccine became available for the 5-to-11-year age group. The initial claims data included 2 137 779 146 observations, representing 31 492 232 individuals from 11 states (Figure). After exclusions, 5 112 722 individuals were included in the overall study population, of whom 2 618 098 were females (51.2%) and 2 491 037 were males (48.7%), with a mean (SD) age of 33.5 (17.6) years. Among these individuals, 5.3% were younger than 5 years, 8.7% were aged 5 to 11 years, 8.1% were aged 12 to 17 years, 12.7% were aged 18 to 25 years, 18.4% were aged 26 to 35 years, 17.4% were aged 36 to 45 years, 16.1% were aged 46 to 55 years, and 13.2% were aged 56 to 64 years.

**Figure.**
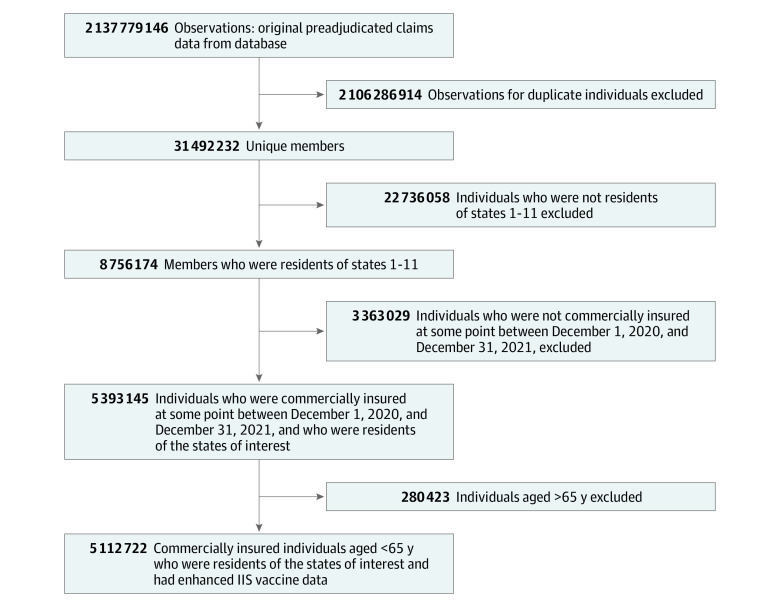
Attrition of the Study Population IIS indicates Immunization Information Systems.

Among those who completed a vaccine series, the greatest number of individuals received their first observed doses in March and April 2021 (eFigure 1 in Supplement 1), and the largest number of individuals received their last observed doses in April and May 2021 (eFigure 2 in Supplement 1). Similar patterns were found across sources of vaccination records.

### Vaccination Coverage Estimates by Data Source

Using claims data, 32.8% of the study population received at least 1 vaccine dose, ranging from 21.6% to 42.8% across 11 states (Table 1). By supplementing claims with IIS data, the overall proportion with at least 1 vaccine dose increased to 48.1%. State 9 had the largest change in estimated vaccinations with the addition of IIS data, increasing from 21.6% (claims data alone) to 46.7% (linked IIS and claims database), whereas State 3 saw the smallest change, increasing from 37.0% to 45.5%.

**Table 1.** Proportion of Individuals by Vaccination Status and Source of Vaccine Data From December 1, 2020, to December 31, 2021

	Total study population	COVID-19 vaccination status, No. (%)			
		Received at least 1 vaccine dose		Completed a vaccine series	
		Claims database^a^	Linked IIS and claims database^b^	Claims database^a^	Linked IIS and claims database^b^
All individuals	5 112 722	1 676 235 (32.8)	2 458 231 (48.1)	1 248 637 (24.4)	2 143 556 (41.9)
State No.					
1	643 602	201 474 (31.3)	316 177 (49.1)	145 137 (22.6)	287 198 (44.6)
2	158 385	47 831 (30.2)	76 820 (48.5)	38 294 (24.2)	68 478 (43.2)
3	1 143 375	422 934 (37.0)	520 249 (45.5)	310 479 (27.2)	404 913 (35.4)
4	696 305	184 312 (26.5)	265 936 (38.2)	135 725 (19.5)	228 643 (32.8)
5	786 234	255 544 (32.5)	401 634 (51.1)	193 105 (24.6)	366 046 (46.6)
6	318 060	136 090 (42.8)	167 745 (52.7)	102 514 (32.2)	144 224 (45.3)
7	330 165	124 739 (37.8)	191 327 (58.0)	101 157 (30.6)	180 397 (54.6)
8	360 267	110 016 (30.5)	179 787 (49.9)	83 987 (23.3)	159 617 (44.3)
9	87 663	18 927 (21.6)	40 901 (46.7)	12 709 (14.5)	36 876 (42.1)
10	219 939	54 303 (24.7)	105 376 (47.9)	39 386 (17.9)	95 468 (43.4)
11	254 098	76 424 (30.1)	133 781 (52.7)	54 735 (21.5)	122 816 (48.3)
Multiple states^c^	114 629	43 641 (38.1)	58 498 (51.0)	31 409 (27.4)	48 880 (42.6)

Abbreviation: IIS, Immunization Information Systems.

^a^ Prior to hierarchical deduplication of vaccine records across IIS and claims databases.

^b^ After hierarchical deduplication of vaccine records across IIS and claims databases.

^c^ There were multiple states listed for a patient among the 11 states of interest.

Similar patterns were observed among individuals with a completed vaccine series using claims only data vs linked IIS and claims data (Table 1). Overall, 24.4% of individuals with commercial insurance completed the vaccine series based on claims data only, which increased to 41.9% using linked IIS and claims data. Similarly, State 9 had the largest increase in vaccination estimate, whereas State 3 had the smallest increase.

### Estimates of the Magnitude of Misclassification 

The CDC and state DOH vaccination estimates were always higher than this study’s linked IIS and claims data estimates. Compared with the CDC estimates, the percentage of underrecording in the linked IIS and claims database varied across 11 states between 15.1% and 47.1% for those with at least 1 vaccine dose (Table 2) and between 12.1% and 45.7% for those with a completed vaccine series (Table 3). For most of the states, the percentage of underrecording in the linked IIS and claims data estimates was higher compared with the CDC vaccination estimates than the state DOH vaccination estimates (Table 2 and Table 3). Compared with the state DOH estimates, the percentage of underrecording in the linked IIS and claims database varied between 10.2% and 39.3% for individuals with at least 1 vaccine dose (Table 2) and between 9.1% and 46.9% for those with a completed vaccine series (Table 3).

**Table 2.** Comparison of the Proportion of Individuals With at Least 1 COVID-19 Vaccine Dose From Linked IIS and Claims Data, CDC, DOH, and Capture-Recapture Analysis

State No.	Age group used for row, y^a^	Linked IIS and claims data vaccination estimate, No. (%)	CDC vaccination estimate, %^b^	% Underrecording^c^	State DOH vaccination estimate, %^b^	% Underrecording^c^	Capture-recapture analysis vaccination estimate, No. (%)^d^	% Underrecording^c^^,^^d^
1	0-64	316 177 (49.1)	59.0	16.8	57.3	14.3	361 638 (56.2)	12.6
2	0-64	76 820 (48.5)	61.5	21.1	NA^e^	NA^e^	91 047 (57.5)	15.7
3	5-64	520 224 (48.3)	86.5	44.2	79.6	39.3	847 815 (74.2)	38.7
4	0-64	265 936 (38.2)	61.5	37.9	59.8	36.1	353 345 (50.7)	24.7
5	5-64	401 549 (53.9)	74.6	27.7	69.5	22.4	485 162 (61.7)	17.2
6	0-64	167 745 (52.7)	89.0	40.8	82.6	36.2	210 931 (66.3)	20.5
7	5-64	191 428 (61.8)	72.8	15.1	68.8	10.2	210 614 (63.8)	9.2
8	5-64	179 899 (52.6)	78.9	33.3	63.7	17.4	225 774 (62.7)	20.4
9	0-64	40 901 (46.7)	88.3	47.1	72.1	35.2	56 322 (64.2)	27.3
10	5-64	105 456 (52.3)	64.8	19.3	65.0	19.5	131 624 (59.8)	19.9
11	12-64	130 385 (59.1)	85.4	30.8	76.9	23.1	162 002 (63.8)	17.5

Abbreviations: CDC, Centers for Disease Control and Prevention; DOH, Department of Health; IIS, Immunization Information Systems; NA, not applicable.

^a^ Some states cover age groups 5 to 64 years or 12 to 64 years because those were the age ranges available for the age-specific DOH data.

^b^ Counts were not provided because they were not consistently available on the websites for the external sources.

^c^ Percentage of underrecording was calculated as the difference between the linked IIS and claims data estimate and the comparison estimate divided by the comparison estimate.

^d^ Estimates for this column were for ages 0 to 64 years. All other columns were restricted to the ages indicated for each row unless otherwise specified.

^e^ Age-specific vaccination estimates were not available on the state website for state 2, precluding age standardization and subsequent inclusion in this table.

**Table 3.** Comparison of the Proportion of Individuals With Completed COVID-19 Vaccine Series From Linked IIS and Claims Data, CDC, DOH, and Capture-Recapture Analysis

State No.	Age group used for row, y^a^	Linked IIS and claims data vaccination estimate, No. (%)	CDC vaccination estimate, %^b^	% Underrecording^c^	State DOH vaccination estimate, %^b^	% Underrecording^c^	Capture-recapture analysis vaccination estimate, No. (%)^d^	% Underrecording^c^^,^^d^
1	0-64	287 198 (44.6)	51.0	12.5	NA^e^	NA^e^	347 045 (53.9)	17.3
2	0-64	68 478 (43.3)	49.7	12.9	NA^e^	NA^e^	84 447 (53.3)	18.8
3	5-64	404 978 (37.6)	69.3	45.7	70.8	46.9	824 768 (72.1)	50.9
4	0-64	228 643 (32.8)	50.7	35.3	NA^e^	NA^e^	341 313 (49.0)	33.1
5	5-64	365 789 (49.1)	65.4	24.9	62.0	20.8	471 860 (60.0)	22.3
6	0-64	144 224 (45.4)	73.1	37.9	71.7	36.7	203 222 (63.9)	29.0
7	5-64	180 277 (58.2)	66.2	12.1	64.0	9.1	202 795 (61.4)	11.1
8	5-64	159 720 (46.7)	63.3	26.2	52.1	10.4	217 222 (60.3)	26.5
9	0-64	36 876 (42.1)	74.5	43.5	65.2	35.4	55 858 (63.7)	33.9
10	5-64	95 374 (47.3)	56.6	16.4	56.1	15.7	125 112 (56.9)	23.7
11	12-64	120 678 (54.7)	76.6	28.6	70.5	22.4	159 174 (62.6)	22.8

Abbreviations: CDC, Centers for Disease Control and Prevention; DOH, Department of Health; IIS, Immunization Information Systems; NA, not applicable.

^a^ Some states cover age groups 5 to 64 years or 12 to 64 years because those were the age ranges available for the age-specific DOH data.

^b^ Counts were not provided because they were not consistently available on the websites for the external sources.

^c^ Percentage of underrecording was calculated as the difference between the linked IIS and claims data estimate and the comparison estimate divided by the comparison estimate.

^d^ Estimates for this column were for ages 0 to 64 years. All other columns were restricted to the ages indicated for each row unless otherwise specified.

^e^ Age-specific vaccination estimates were not available on the state websites for states 1, 2, and 4, which precluded age standardization and subsequent inclusion in this table.

Using a capture-recapture analysis, we estimated that 14.5% of individuals from the study population (n = 739 128) were misclassified as unvaccinated because they had no vaccine records in the IIS or claims database, but this analysis suggested they had received a vaccine dose (not tabulated). This misclassification was associated with a decrease in the estimated number of unvaccinated individuals from 2 654 491 to 1 915 363 and an increase in the proportion of individuals who received at least 1 vaccine dose to 62.5% (not tabulated). Compared with capture-recapture estimates, the percentage of underrecording in the linked IIS and claims database varied across states between 9.2% and 38.7% for individuals who received at least 1 vaccine dose and between 11.1% and 50.9% for those who completed a vaccine series (Table 2 and Table 3).

### Sensitivity Analysis

By restricting the analysis to individuals with continuous insurance coverage during the study period, the vaccination coverage estimates increased from 48.1% to 55.5% for individuals who received at least 1 vaccine dose and from 41.9% to 49.1% for those with a completed vaccine series. Additionally, by restricting the analysis to individuals who only had health care claims from a single state, the vaccination coverage estimates increased from 48.1% to 48.8% for those who received at least 1 vaccine dose and from 41.9% to 43.0% for those who completed a vaccine series.

## Discussion

We found that a linked IIS and claims database was associated with substantial improvement in the ascertainment of COVID-19 vaccine administration compared with a claims database alone. However, even with the linked IIS and claims data, the ascertainment of COVID-19 vaccination was likely incomplete given the misclassification estimates obtained from comparisons with CDC data, state DOH data, and capture-recapture analysis.

The proportion of individuals with at least 1 vaccine dose of a COVID-19 vaccine increased by 47.1% when we augmented the claims data with IIS data. Without the incorporation of IIS data into claims data, considerable misclassification of vaccination status would occur, particularly vaccinated individuals being counted as unvaccinated. This misclassification could limit statistical power for vaccine safety studies because of smaller sample sizes of vaccinees, which could impair the ability to detect rare adverse events. Additionally, this misclassification is associated with COVID-19 vaccines appearing less effective in the context of effectiveness studies because some individuals in the unvaccinated group could have received the vaccine, reducing the contrast between the vaccinated and unvaccinated groups.^2^ Recognizing the importance of mitigating misclassification for vaccine research, the CDC’s Vaccine Safety Datalink collaborative undertook an effort to link IIS data to electronic health record data.^7^

The linked IIS and claims data estimates were 9.2% to 50.9% lower than the capture-recapture–adjusted estimates, varying by state. An assumption of the capture-recapture analysis was that the capture mechanisms for IIS and claims vaccine records were independent.^8^ This assumption was likely not satisfied because vaccination providers who submitted a claim were also asked to report to IIS repositories. Because the assumption of independence between data sources was violated, the calculation underestimated the misclassification of vaccination.

State-specific linked IIS and claims data estimates were 12.1% to 47.1% lower than age-standardized CDC estimates. The CDC estimates potentially overestimated vaccination because the CDC receives vaccine records on a rolling basis from multiple sources that could include duplicates.^9^ The CDC vaccination data include vaccine records not only from IIS databases but also from territory, tribe, and local entities; retail pharmacies; long-term care facilities; dialysis centers participating in the Federal Dialysis Center Program; Federal Emergency Management Agency partner sites; Health Resources and Services Administration partner sites; and other federal entity facilities.^9^ Vaccine records that are submitted to the CDC are deidentified, have a unique identification number, and are not always linkable. Also contributing to overestimation is that the population the CDC uses as the denominator in calculations may sometimes be based on outdated US Census counts.^8,9^ Although we age-standardized the CDC estimates to mimic the population profile, this method may not have fully accounted for age differences between populations due to wide age groupings (for the adult population), and we did not control for payer and underlying health conditions.

State-specific linked IIS and claims data estimates were 9.1% to 46.9% lower than age-standardized DOH estimates. A strength of the DOH data is that they can more easily link multiple doses to an individual resident of the state than the CDC estimates, which make DOH estimates not as susceptible to duplicates. Granular age stratifications are typically more available for DOH estimates than CDC estimates (eTable 1 in Supplement 1), allowing for better age standardization to the study population. However, as the IIS data are a component in the linked vaccination database and are generally the basis of DOH estimates, the DOH estimates may not add many missed vaccinations compared with the linked IIS and claims data estimates because they use the same underlying source data. Moreover, IIS data presented on DOH websites were not specific to the commercial insurance population and included vaccination information on individuals without insurance or those covered by other payers, such as Medicaid and other commercial plans, which could explain the differences between the estimates. Beyond informing potential misclassification, the comparison to DOH data serves as an internal validation of the study data because they were both generally derived from the same IIS data source.

Estimates of at least 1 vaccine dose increased from 48.1% to 55.5% when we restricted the analysis to individuals with continuous insurance enrollment. This increase in the vaccination estimates when restricting to complete claims data further underscores the value of using a variety of data sources (ie, either data source alone missed vaccinations) for estimating vaccination coverage. Additionally, the underrecording estimates were likely overestimates for studies that were restricted to individuals with continuous coverage and would result in larger bounds for quantitative bias analyses than may be appropriate. Alternatively, restricting to individuals with health care claims from only 1 state did not appreciably alter the vaccination estimates, suggesting minimal association between cross-state migration and vaccination coverage estimates in this study.

### Limitations

This study has some limitations. First, the number of individuals who completed a vaccine series may be underestimated due to incomplete second vaccine dose records that were censored by the data cutoff (December 31, 2021); this underestimation most likely affected the younger age groups based on the vaccine rollout. Two-dose COVID-19 vaccines for the 5-to-11-year age group were authorized by the FDA in late October 2021. Second, IIS data had limitations that were factors in missed vaccine records. Although a COVID-19 vaccination reporting requirement was in place, some vaccination providers submitted records directly to the CDC, and there was variation in consent mandates (eg, opt-in for all ages and opt-in for children) across states. There may be data linkage issues between the submitting vaccination provider and the IIS database, such as misspelling of the vaccinee’s name, wrong date of birth, or failure to report the vaccination. Furthermore, each IIS repository used an IIS-specific linkage algorithm to match individuals on a roster to vaccines that were administered in their jurisdiction, which may contribute to differences in matching quality. Third, these study results are applicable to estimating COVID-19 vaccine coverage for the commercially insured population in 11 states and might not be applicable to estimating coverage for other vaccines; other payers, uninsured people, or dually insured populations; or other states in the US.

## Conclusions 

In this cohort study of individuals younger than 65 years with commercial insurance in 11 states, using medical claims alone was associated with substantial underestimation of COVID-19 vaccine coverage during the study period. Incorporating IIS vaccination records into claims data increased the number of individuals who were identified as vaccinated, but underrecording remained. These findings not only underscored the value of using a variety of data sources and the importance of accounting for health coverage gaps using claims data alone but also highlighted the difficulty of estimating vaccination coverage without a universal health identifier. Additionally, we quantified the ascertainment of vaccine administration using linked IIS and claims databases and informed strategies to account for incomplete vaccine data in safety and effectiveness studies. To support public health, vaccination providers, health systems, and IIS users across the US should strive for improvements in reporting of vaccination data to IIS databases, data exchanges, and IIS data infrastructures, which would allow frequent updates of vaccine status for all US individuals and all vaccines.

## Notes

Supplement 1. **eTable 1.** Refresh Frequency of IIS Data in the IIS Repositories, Last Date of Service in the IIS Repositories, Date of CDC and DOH Data, and Age Groupings for Age-Standardization of DOH Data **eTable 2.** Summary of Individual IIS Linkage Algorithms **eTable 3.** Steps for IIS and Claims Vaccine Record Cleaning **eTable 4.** Variable Definitions **eMethods 1.** Additional Information on Age Standardization **eMethods 2.** Additional Information on the Capture-Recapture Method **eTable 5.** Characteristics of the Study Population, Total and Among Those With At Least One Dose of COVID-19 Vaccine by Source of Vaccine Record: December 1, 2020 – December 31, 2021 **eTable 6.** Characteristics of the Study Population, Total and Among Those With Completed COVID-19 Vaccine Series by Source of Vaccine Record: December 1, 2020 – December 31, 2021 **eFigure 1.** Number of Individuals by Month of Administration of First Observed COVID-19 Dose and Data Source **eFigure 2.** Number of Individuals by Month of Administration of Last Observed COVID-19 Dose and Data Source

Supplement 2.
Data Sharing Statement
