# Errors in Byline and Affiliation

**DOI:** 10.1001/jamanetworkopen.2023.21247

**Published:** 2023-06-13

In the Original Investigation “Use of Immunization Information Systems in Ascertainment of COVID-19 Vaccinations for Claims-Based Vaccine Safety and Effectiveness Studies,”^1^ published May 16, 2023, the name of author Cindy K. Zhou, PhD, has been corrected in the byline, and her affiliations have been updated to reflect her employment at the US Food and Drug Administration during the conduct of the study. They are now given as “Center for Biologics Evaluation and Research, US Food and Drug Administration, Silver Spring, Maryland” and “Now with Clinical Safety and Risk Management, Moderna, Cambridge, Massachusetts.” This article has been corrected.^1^
