# Supplementary material for: Use of Immunization Information Systems in Ascertainment of COVID-19 Vaccinations for Claims-Based Vaccine Safety and Effectiveness Studies
Source: JAMA Netw Open. 2023 May 16;6(5):e2313512. doi: 10.1001/jamanetworkopen.2023.13512 (PMC10189561; doi:10.1001/jamanetworkopen.2023.13512)
Supplement: Supplement 1. — eTable 1. Refresh Frequency of IIS Data in the IIS Repositories, Last Date of Service in the IIS Repositories, Date of CDC and DOH Data, and Age Groupings for Age-Standardization of DOH Data eTable 2. Summary of Individual IIS Linkage Algorithms eTable 3. Steps for IIS and Claims Vaccine Record Cleaning eTable 4. Variable Definitions eMethods 1. Additional Information on Age Standardization eMethods 2. Additional Information on the Capture-Recapture Method eTable 5. Characteristics of the Study Population, Total and Among Those With At Least One Dose of COVID-19 Vaccine by Source of Vaccine Record: December 1, 2020 – December 31, 2021 eTable 6. Characteristics of the Study Population, Total and Among Those With Completed COVID-19 Vaccine Series by Source of Vaccine Record: December 1, 2020 – December 31, 2021 eFigure 1. Number of Individuals by Month of Administration of First Observed COVID-19 Dose and Data Source eFigure 2. Number of Individuals by Month of Administration of Last Observed COVID-19 Dose and Data Source [file jamanetwopen-e2313512-s001.pdf]

Supplemental Online Content

Schneider KL, Bell EJ, Zhou CK, et al. Use of Immunization Information Systems in ascertainment of COVID-19 vaccinations for claims-based vaccine safety and effectiveness studies. *JAMA Netw Open*. 2023;6(5):e2313512. doi:10.1001/jamanetworkopen.2023.13512

eTable 1. Refresh Frequency of IIS Data in the IIS Repositories, Last Date of Service in the IIS Repositories, Date of CDC and DOH Data, and Age Groupings for Age-Standardization of DOH Data

eTable 2. Summary of Individual IIS Linkage Algorithms

eTable 3. Steps for IIS and Claims Vaccine Record Cleaning

eTable 4. Variable Definitions

eMethods 1. Additional Information on Age Standardization

eMethods 2. Additional Information on the Capture-Recapture Method

eTable 5. Characteristics of the Study Population, Total and Among Those With At Least One Dose of COVID-19 Vaccine by Source of Vaccine Record: December 1, 2020 – December 31, 2021

eTable 6. Characteristics of the Study Population, Total and Among Those With Completed COVID-19 Vaccine Series by Source of Vaccine Record: December 1, 2020 – December 31, 2021

eFigure 1. Number of Individuals by Month of Administration of First Observed COVID-19 Dose and Data Source

eFigure 2. Number of Individuals by Month of Administration of Last Observed COVID-19 Dose and Data Source

This supplemental material has been provided by the authors to give readers additional information about their work.

© 2023 Schneider KL et al. *JAMA Network Open*

eTable 1. Refresh frequency of IIS data in the IIS repositories, last date of service in the IIS repositories, date of CDC and DOH data, and age groupings for age-standardization of DOH data

State IIS	Refresh frequency	Last observed date of service	Date of CDC data[a]	Date of DOH data[a]	DOH groups for age-standardization
State 1	Monthly	11/21/21	11/3/21	11/21/21	< 20, 20-34, 35-44, 45-54, 55-64
State 2	Monthly	12/02/21	1/13/22	N/A	N/A
State 3	Monthly	12/31/21	1/13/22	1/11/22	5-11, 12-17, 18-45, 50-64
State 4	Semi-monthly	12/3/21	1/13/22	1/11/22	0-4, 5-9, 10-14, 15-19, 20-24, 25-34, 35-44, 45-54, 55-64
State 5	Weekly	12/31/21	1/13/22	1/11/22	5-11, 12-17, 18-64
State 6	Weekly	12/31/21	1/13/22	1/11/22	0-4, 5-11, 12-15, 16-19, 20-29, 30-39, 40-49, 50-64
State 7	Weekly	12/31/21	1/13/22	1/17/22	5-11, 12-15, 16-17, 18-49, 50-64
State 8	Monthly	12/23/21	1/13/22	1/14/22	5-9, 10-14, 15-19, 20-24, 25-29, 30-34, 35-39, 40-44, 45-49, 50-54, 55-59, 60-64
State 9	Daily	12/30/21	1/13/22	1/01/22	0-4, 5-9, 10-14, 15-18, 19-24, 25-29, 30-39, 40-49, 50-59, 60-69
State 10	Monthly	11/23/21	11/3/21	11/23/21	5-11, 12-18, 19-29, 30-39, 40-49, 50-59, 60-69
State 11	Monthly	12/17/21	1/13/22	12/15/21	12-15, 16-17, 18-34, 35-49, 50-64

Abbreviations: CDC = Centers for Disease Control and Prevention, DOH = department of health, IIS = Immunization Information System, N/A = not applicable

[a] Dates for the CDC and DOH data were chosen to most closely match the last date of service of the IIS data.

Notes: CDC age groups used for age standardization were: 0-4 years, 5-11 years, 12-17 years, and 18-64 years. N/A = age-specific vaccination estimates were not available on the state website for State 2.

© 2023 Schneider KL et al. *JAMA Network Open*

eTable 2. Summary of individual IIS linkage algorithms and match percentage

State IIS	Description of Linkage	Match Percentage[a]
State 1	Linkage variables: first, middle and last name; date of birth; gender; medical record number. If multiple matches, use address, phone number, guardian name.	42.8%
State 2	Linkage variables: first and last name; date of birth. If multiple matches, use WebIZ ID, social security number, middle name, gender, suffix, phone number.	30.9%
State 3	Linkage variables: member ID; first and last name; date of birth.	18.9%
State 4	Linkage variables: first and last name; date of birth; chart ID.	14.8%
State 5	Linkage variables: first and last name; date of birth. If multiple matches, use address.	39.7%
State 6	Linkage variables: first, middle and last name; address; date of birth; phone number. Only returns data for patients with 1:1 matches.	52.4%
State 7	Linkage variables: first and last name; date of birth. Probabilistic matching and only returns 1:1 matches.	49.1%
State 8	Linkage variables: first and last name; date of birth. If multiple matches, use WebIZ ID, social security number, middle name, gender, suffix.	25.1%
State 9	Linkage variables: first and last name; date of birth. Deterministic matching.	43.1%
State 10	Linkage variables: first, middle, and last name; suffix; date of birth; gender; social security number; mother's first and maiden name; address; phone number. Mix of probabilistic and deterministic matching.	12.1%
State 11	Linkage variables: first and last name; date of birth; gender; address; phone. Deterministic matching.	43.6%

Abbreviations: ID = identification number, IIS = Immunization Information System

[a] Match percentage is calculated as the membership count divided by the number of records returned from the IIS.

© 2023 Schneider KL et al. *JAMA Network Open*

eTable 3. Steps for IIS and claims vaccine record cleaning

Step #	Data Cleaning Step Description
1	**Linked IIS vaccination data to pre-adjudicated claims**
2	**Transformed claims vaccine data (CPT®, NDC, HCPCS codes, and ICD-10 procedure codes) to CVX codes and dose number for the purposes of deduplication, but kept the original vaccine codes for analyses**
3	**Restricted data to COVID-19 vaccine codes within appropriate time frame:**<br>1) Restricted to COVID-19 vaccine codes: CVX codes 207 (mRNA-1273), 208 (BNT162b2), 212 (Ad26.COV2.S), 213 (unknown vaccine type), 217 (BNT162b2 tris-sucrose formulation for ages 12+), 218 (BNT162b2 tris-sucrose formulation for ages 5 to < 12), and 219 (BNT162b2 tris-sucrose formulation for ages 2 to < 5)<br>2) Removed records with blank member ID or date of service<br>3) Removed records with dates before vaccine authorization:<br>If CVX Code = 207, then removed records prior to 18 December 2020<br>If CVX Code = 208, then removed records prior to 11 December 2020<br>If CVX Code = 212, then removed records prior to 27 February 2021<br>If CVX Code = 213, then removed records prior to 11 December 2020<br>If CVX Code = 217, then removed records prior to 03 September 2021<br>If CVX Code = 218, then removed records prior to 29 October 2021<br>If CVX Code = 219, then removed records because it was not yet authorized
4	**Deduplicated vaccine records within each data source,[a] prioritized non-missing dose number**<br>1) Within pre-adjudicated and IIS data sources, records were grouped by member ID, date of vaccine, CVX vaccine code, and dose number. Observations with duplicates of all four variables were removed.<br>2) Records with the same member ID, date of service, and CVX code were then further deduplicated, with priority on keeping records with non-missing dose number. In other words, we only removed records that had a missing dose number when there was another record with a non-missing dose number, with the same CVX and record date for that member. But if there were two records on the same date, same CVX, with different non-missing doses (e.g., dose 3 and dose 2), both were kept.
5	**Deduplication of vaccine records, prioritized by data source**<br>1) Set up data source hierarchy rules:<br>If the source was commercial pharmacy claims, then priority was set to '4'<br>If the source was commercial medical claims, then priority was set to '3'<br>If the source was IIS, then priority was set to '2'<br>If the source was Medicare, then priority was set to '1'<br>2) Records were grouped by member ID, date of service, and CVX vaccine code. Then, these records were deduplicated, keeping only one record with the same date of service and CVX vaccine code per member ID, with priority on keeping the record highest in the data source hierarchy (4 being highest).

Abbreviations: CPT = Current Procedural Terminology, CVX = Vaccine administered code, HCPCS = Healthcare Common Procedure Coding System, ICD = International Classification of Diseases, ID = Identification number, IIS = Immunization Information System, NDC = National Drug Code

[a] IIS data were treated as a single source.

© 2023 Schneider KL et al. *JAMA Network Open*

eTable 4. Variable definitions

Variables	Definitions
State of residence	Based on pre-adjudicated claims data. Claims were searched between 01 January 2017 and 31 December 2021 for all associated values. State was set to “Multiple States” if there was more than one state identified per patient.
Age	Based on pre-adjudicated claims and IIS data. Age calculated on 31 December 2021. When member records indicated differing dates of birth or, when both were missing, it was set to unknown.
Sex	Based on pre-adjudicated claims and IIS data. When member records indicated differing sex or, when both were missing, it was set to unknown.

Abbreviations: IIS = Immunization Information System

© 2023 Schneider KL et al. *JAMA Network Open*

eMethods 1. Additional information on age standardization

We obtained age-specific vaccination estimates from Centers for Disease Control and Prevention (CDC) and department of health (DOH), and respectively standardized the CDC and DOH estimates to the age distribution of our commercial population residing in each state. Age-stratified vaccination estimates were available online for all states except State 2. Some DOH provided age-specific vaccination coverage estimates for a subset of the non-elderly population (e.g., vaccination coverage estimates for individuals aged 5–64 or 12–64 years), while others provided vaccination estimates for the entire non-elderly population (0–64 years). Additional information on age bands for standardization and time periods of data can be found in eTable 1.

© 2023 Schneider KL et al. *JAMA Network Open*

eMethods 2. Additional information on the capture-recapture method

The capture-recapture analysis estimated the number of people who would be misclassified as unvaccinated if vaccination status was based on a combination of claims and Immunization Information System (IIS) vaccine records. Briefly, capture-recapture analysis uses counts from two independent data sources to estimate the number of individuals who were not captured by either of the data sources. The assumptions of capture-recapture are: 1) independence of capture by the two data sources, 2) all individuals have the same probability of being captured, 3) no migration in or out of the catchment area, and 4) no loss of identifier. Although these assumptions are for the most part untestable, the likelihood that they are being met and the impact on results if not met is considered in the discussion section. In this analysis, the two data sources were vaccine records from IIS data and pre-adjudicated claims data for our study population.

Under the assumption of independent samples, the maximum likelihood estimator of the odds ratio measuring the association between the two samples, $\frac{ad}{bc}$, should equal 1.0. Since the values in cells *a*, *b*, and *c* are known from the source of the two samples (assuming individuals have a unique identifier so those common to the two samples can be identified), the unknown value is *d*. Rearranging the terms of the equation to solve for *d* yields: $d = \frac{bc}{a}$. As illustrated in the table, by deriving cell d using the factors a, b, and c, we estimated the number of individuals in our study population who were truly vaccinated but misclassified as unvaccinated because a vaccine record was not captured in pre-adjudicated claims or IIS. We then re-calculated vaccination estimates, re-classifying the number identified in cell *d* as vaccinated for this analysis.

2x2 table used for capture-recapture analysis

		**Vaccine record identified in IIS?**	
		**Yes**	**No**
**Vaccine record identified in claims?**	**Yes**	a	b
	**No**	c	d

© 2023 Schneider KL et al. *JAMA Network Open*

eTable 5. Characteristics of the study population, total and among those with at least one dose of COVID-19 vaccine by source of vaccine record: December 1, 2020 – December 31, 2021.

Characteristic	Total study population (No. = 5,112,722)	Members with at least one dose in pre-adjudicated claims[a] (No. = 1,676,235)	Members with at least one dose in IIS[a] (No. = 1,643,733)
	% (No.)	% (No.)	% (No.)
**State**			
State 1	12.6% (643,602)	12.0% (201,474)	15.8% (258,991)
State 2	3.1% (158,385)	2.9% (47,831)	3.7% (61,074)
State 3	22.4% (1,143,375)	25.2% (422,934)	11.8% (194,184)
State 4	13.6% (696,305)	11.0% (184,312)	10.4% (170,626)
State 5	15.4% (786,234)	15.2% (255,544)	18.8% (308,675)
State 6	6.2% (318,060)	8.1% (136,090)	5.4% (89,216)
State 7	6.5% (330,165)	7.4% (124,739)	9.9% (163,311)
State 8	7.0% (360,267)	6.6% (110,016)	8.3% (136,081)
State 9	1.7% (87,663)	1.1% (18,927)	2.0% (33,096)
State 10	4.3% (219,939)	3.2% (54,303)	5.3% (86,942)
State 11	5.0% (254,098)	4.6% (76,424)	6.6% (108,579)
Multiple states[b]	2.2% (114,629)	2.6% (43,641)	2.0% (32,958)
**Sex**			
Female	51.2% (2,618,098)	51.7% (866,261)	52.2% (858,337)
Male	48.7% (2,491,037)	48.3% (809,051)	47.6% (782,121)
Unknown	0.1% (3,587)	0.1% (923)	0.2% (3,275)
**Age[c]**			
< 5 years	5.3% (273,419)	0.0% (18)	0.1% (1,220)
5 – 11 years	8.7% (443,138)	4.8% (80,538)	0.5% (8,654)
12 – 17 years	8.1% (413,499)	7.2% (121,160)	7.4% (122,242)
18 – 25 years	12.7% (649,067)	11.7% (195,367)	9.9% (162,091)
26 – 35 years	18.4% (941,101)	18.9% (317,135)	18.0% (295,873)
36 – 45 years	17.4% (890,290)	20.1% (336,142)	22.2% (364,369)
46 – 55 years	16.1% (824,346)	19.7% (329,839)	22.2% (364,989)
56 – 64 years	13.2% (676,105)	17.6% (295,635)	19.7% (324,293)
**Type of vaccine**			
BNT162b2 (Pfizer-BioNTech)	N/A	61.4% (1,028,958)	59.0% (970,155)
mRNA-1273 (Moderna)	N/A	28.6% (479,607)	29.9% (491,029)
Ad26.COV2.S (Janssen)	N/A	4.1% (68,858)	5.3% (86,307)
Mixed series/Unknown[d]	N/A	5.9% (98,812)	5.9% (96,242)

Abbreviations: IIS = Immunization Information System, N/A = not applicable

[a] Prior to deduplication of vaccine records across sources (i.e., IIS, pre-adjudicated claims).

[b] There were multiple states listed for a member, among the eleven states of interest.

[c] Age was missing for < 0.1% for all three groups.

[d] Two records that were different brands/types of vaccines were defined as mixed series (e.g., one dose of BNT162b2 and one dose of mRNA-1273).

Notes: Reporting column percentages. Characteristics of the total study population for type of vaccine are labeled as N/A because these are based on the total study population, regardless of vaccine.

© 2023 Schneider KL et al. *JAMA Network Open*

eTable 6: Characteristics of the study population, total and among those with completed COVID-19 vaccine series by source of vaccine record: December 1, 2020 – December 31, 2021.

Characteristic	Total study population (N = 5,112,722)	Members with a completed vaccine series in pre-adjudicated claims[a] (N = 1,248,637)	Members with a completed vaccine series in IIS[a] (N = 1,493,706)
	% (No.)	% (No.)	% (No.)
**State**			
State 1	12.6% (643,602)	11.6% (145,137)	16.2% (242,188)
State 2	3.1% (158,385)	3.1% (38,294)	3.7% (54,630)
State 3	22.4% (1,143,375)	24.9% (310,479)	9.7% (145,339)
State 4	13.6% (696,305)	10.9% (135,725)	10.2% (151,699)
State 5	15.4% (786,234)	15.5% (193,105)	19.5% (291,153)
State 6	6.2% (318,060)	8.2% (102,514)	5.6% (83,405)
State 7	6.5% (330,165)	8.1% (101,157)	10.6% (157,766)
State 8	7.0% (360,267)	6.7% (83,987)	8.2% (122,550)
State 9	1.7% (87,663)	1.0% (12,709)	2.1% (31,153)
State 10	4.3% (219,939)	3.2% (39,386)	5.5% (81,552)
State 11	5.0% (254,098)	4.4% (54,735)	6.9% (103,170)
Multiple states[b]	2.2% (114,629)	2.5% (31,409)	1.9% (29,101)
**Sex**			
Female	51.2% (2,618,098)	51.4% (641,978)	52.3% (781,031)
Male	48.7% (2,491,037)	48.5% (606,161)	47.5% (709,654)
Unknown	0.1% (3,587)	0.0% (498)	0.2% (3,021)
**Age**[c]			
< 5 years	5.3% (273,419)	0.0% (< 11)	0.1% (1,083)
5 – 11 years	8.7% (443,138)	4.2% (52,979)	0.5% (7,055)
12 – 17 years	8.1% (413,499)	8.0% (100,304)	7.2% (108,205)
18 – 25 years	12.7% (649,067)	11.7% (146,150)	9.6% (143,794)
26 – 35 years	18.4% (941,101)	18.7% (233,084)	17.8% (266,057)
36 – 45 years	17.4% (890,290)	20.0% (249,629)	22.1% (330,782)
46 – 55 years	16.1% (824,346)	19.7% (246,537)	22.4% (335,225)
56 – 64 years	13.2% (676,105)	17.6% (219,678)	20.2% (301,503)
**Type of vaccine**			
BNT162b2 (Pfizer-BioNTech)	N/A	60.5% (755,558)	58.5% (873,150)
mRNA-1273 (Moderna)	N/A	28.0% (349,788)	29.6% (442,135)
Ad26.COV2.S (Janssen)	N/A	5.5% (68,858)	5.8% (86,305)
Mixed/Unknown	N/A	6.0% (74,433)	6.2% (92,116)

Abbreviations: IIS = Immunization Information System, N/A = not applicable
[a] Prior to deduplication of vaccine records across sources (i.e., IIS, pre-adjudicated claims).
[b] There were multiple states listed for a patient, among the eleven states of interest.
[c] Age was missing for < 0.1% for all three groups.
Notes: Reporting column percentages. Characteristics of the total study population for brand of vaccine are labeled as N/A because these are based on enrollment data before identification of vaccine status in claims or IIS data.

© 2023 Schneider KL et al. *JAMA Network Open*

eFigure 1. Number of individuals by month of administration of first observed COVID-19 dose and data source.

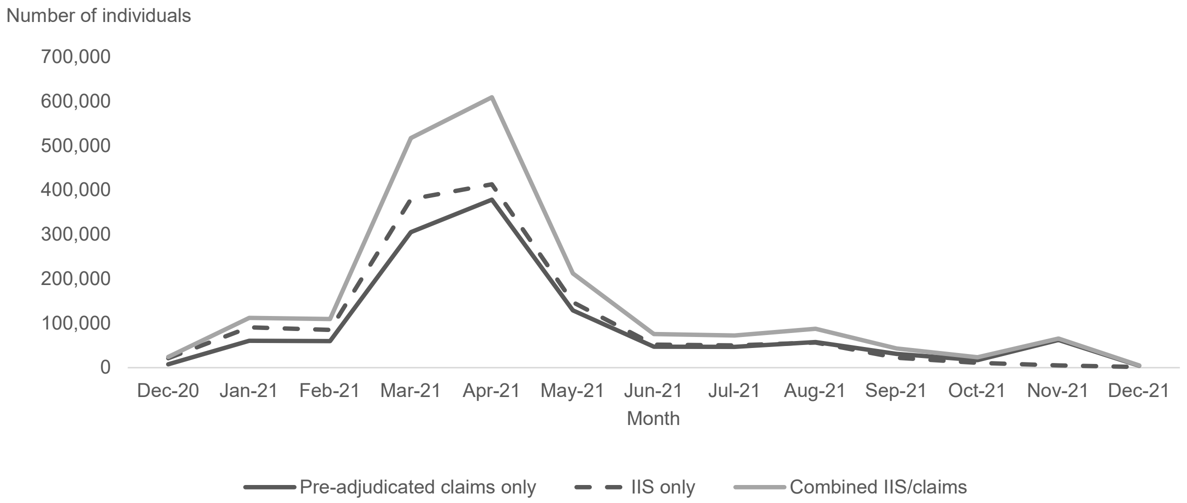

Abbreviations: IIS = Immunization Information System
Note: Only including those individuals who completed a vaccine series, so eligible for at least two doses. Eleven percent of individuals are excluded because they had conflicting dates of service for the first dose in the IIS and pre-adjudicated claims.

© 2023 Schneider KL et al. *JAMA Network Open*

eFigure 2. Number of individuals by month of administration of last observed COVID-19 dose and data source.

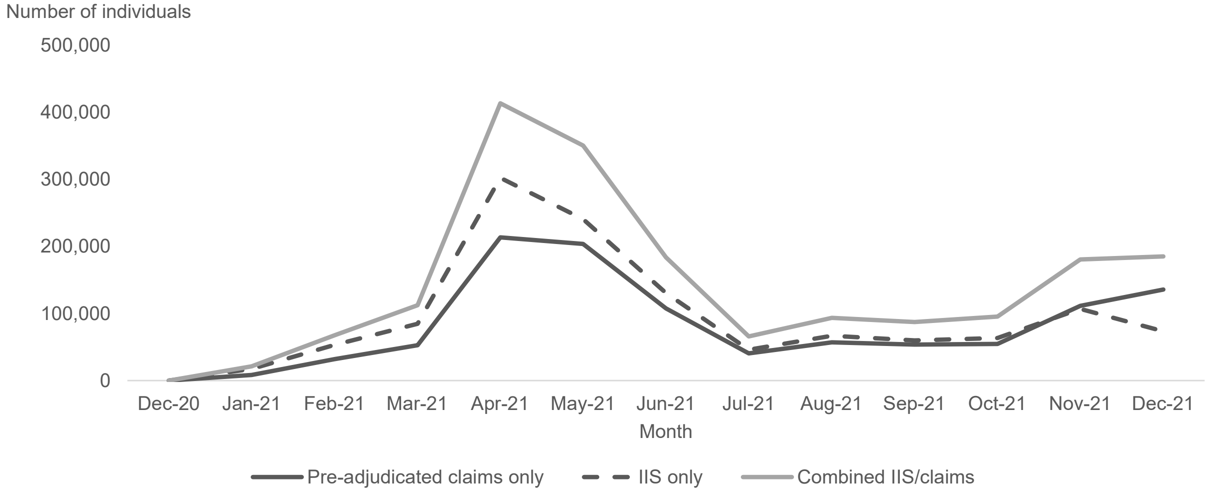

Abbreviations: IIS = Immunization Information System
Note: Only including those individuals who completed a vaccine series, so eligible for at least two doses. Eleven percent of individuals are excluded because they had conflicting dates of service for the first dose in the IIS and pre-adjudicated claims.

© 2023 Schneider KL et al. *JAMA Network Open*
