# Supplementary material for: Use of Immunization Information Systems in Ascertainment of COVID-19 Vaccinations for Claims-Based Vaccine Safety and Effectiveness Studies
Source: JAMA Netw Open. 2023 May 16;6(5):e2313512. doi: 10.1001/jamanetworkopen.2023.13512 (PMC10189561; doi:10.1001/jamanetworkopen.2023.13512)
Supplement: Supplement 2. — Data Sharing Statement [file jamanetwopen-e2313512-s002.pdf]

Data Sharing Statement

Schneider. Use of Immunization Information Systems in Ascertainment of COVID-19 Vaccinations for Claims-Based Vaccine Safety and Effectiveness Studies. *JAMA Netw Open*. Published May 16, 2023. doi:10.1001/jamanetworkopen.2023.13512

Data
Data available: No

Additional Information
Explanation for why data not available: The medical and pharmacy claims data are proprietary data to Optum, and the data use agreements made with IIS limit the data to Optum's use.
